# Hepatic Morphological Lesions and Blood Biochemical Evidence of Impaired Metabolism in Wild European Moose (*Alces alces*) Naturally Infected With *Parafasciolopsis fasciolaemorpha*

**DOI:** 10.1155/vmi/2171927

**Published:** 2026-04-17

**Authors:** Baiba Bergmane, Alina Klavina, Maksims Zolovs, Dace Gorbacevska

**Affiliations:** ^1^ Faculty of Veterinary Medicine, Latvia University of Life Sciences and Technologies, Jelgava, Latvia, llu.lv; ^2^ Statistics Unit, Riga Stradins University, Riga, Latvia, rsu.lv; ^3^ Institute of Life Sciences and Technology, Daugavpils University, Daugavpils, Latvia, du.lv

**Keywords:** blood biochemistry, hepatobiliary alterations, moose, parafasciolopsosis

## Abstract

Liver flukes may cause significant pathological changes in domestic and wild ruminants. One such parasite, *Parafasciolopsis fasciolaemorpha*, reported in European moose (*Alces alces*), still requires detailed investigation to better understand its pathogenesis and impact on host health. This study aimed to evaluate the gross, histopathological, and biochemical alterations associated with natural *P. fasciolaemorpha* infection in wild moose inhabiting forests throughout Latvia. Livers from 63 animals were examined, of which 15 (24%) were infected. Infected livers exhibited bile duct thickening and cavities containing trematodes and necrotic debris. Histologically, lesions in infected moose were observed in 82% of examined microscopic fields for portal inflammatory infiltration, 60% for portal fibrosis, and 47% for bile duct proliferation, compared with low frequencies in uninfected animals. Generalized linear mixed models confirmed significant associations between infection and portal inflammation, fibrosis, and bile duct proliferation, whereas bile duct mucous gland hyperplasia did not differ significantly from uninfected moose. Biochemically, infected moose showed reduced serum urea and amylase concentrations, while other hepatic enzyme values did not differ from uninfected controls, indicating mild impairment of hepatic function and possible pancreatic involvement. Fecal examination confirmed the presence of *P. fasciolaemorpha* eggs and revealed frequent co‐infections with gastrointestinal parasites. These findings demonstrate that *P. fasciolaemorpha* infection in moose is associated with distinctive hepatic lesions and subtle but measurable biochemical alterations, expanding knowledge of this understudied trematode and emphasizing its importance in veterinary pathology and wildlife health.

## 1. Introduction

Empirical studies have demonstrated the ecological and management significance of host and parasite relationship in regulation of the wildlife conservation and population, as parasitic infections can reduce survival, reproductive success, and body condition in wild mammals including cervids thereby influencing population dynamics [1]. In cervids, parasitic flatworms belonging to the phylum *Platyhelminthes*, class *Trematoda*, order *Digenea*, and family *Fasciolidae* include flukes which are among the most important helminths affecting wild and domestic ungulates, including hepatic parasites from genera such as *Fasciola*, *Fascioloides*, and *Parafasciolopsis* [2]. Most significant parasites of this group are *Fasciola hepatica* and *Fasciola gigantica*–major liver flukes of domestic ruminants and occasionally wildlife and humans, as well as *Fascioloides magna*–gigant liver fluke described in domestic and wild ruminants [3]. In contrast to other liver flukes, *Parafasciolopsis* spp. is seldom encountered and is considered an uncommon parasite in most parasitological surveys. Its species *Parafasciolopsis fasciolaemorpha* is distinguished by its adaptation to cervid hosts, particularly European moose, also known as elk (*Alces alces*) [4–6] and also been found in other wild ruminants such as red deer [7], roe deer [8], and European bison [5, 9]. The authors refer also other studies, much of the early references not readily accessible through contemporary scientific sources.

*Parafasciolopsis fasciolaemorpha* was first identified by Ejsmont in the early 20th century from moose in Eastern Europe. It follows a typical digenean trematode life cycle, using great ramshorn snails *Planorbarius corneus* as intermediate hosts and wild ruminants, primarily moose as definitive hosts. The disease parafasciolopsosis caused by this parasite may be particulary common in animals that inhabit wetland‐rich forests of Central and Eastern Europe, where the fluke’s life cycle is supported by moose foraging behavior and snail ecology [4, 10].

The parasite *P. fasciolaemorpha* is a small, leaf‐shaped liver fluke (2.9–7.5 × 1.1–2.5 mm) with prominet anterior and narrowed posterior end, a spiked tegument, prominent oral and ventral suckers for tissue adhesion [11]. Its tegumental and digestive structures, including microvillous intestinal epithelium, support efficient nutrient uptake from bile [12, 13]. *Parafasciolopsis fasciolaemorpha* as other liver trematodes is associated with biliary disease [11, 14, 15]. During light to moderate infections, the adult flukes are localized within the intrahepatic bile ducts, where they attach to the mucosal lining and begin their reproductive cycle. However, in severe infections, the parasite extends beyond the bile ducts and has also been observed in the extrahepatic ducts, gallbladder, and occasionally in the duodenum and pancreatic ducts [4]. Heavy infections associated with significant hepatic damage, including hepatomegaly, bile duct obstruction, and the formation of pseudocysts that contain adult flukes, numerous eggs, necrotic debris, and fluid [4, 11]. Histopathological changes include ductal hyperplasia, chronic inflammatory infiltrates, and fibrosis, which in chronic cases may progress to septal cirrhosis [11]. Eggs are typically deposited in the bile ducts for excretion through feces, but in severe cases, they are also found in large numbers within the hepatic pseudocysts, trapped alongside adult trematodes [11]. The eggs are short, golden to grey, and relatively broad, and they can be differentiated from those of *Fasciola hepatica* and *Paramphistomum cervi* by their more compact shape, smoother surface, and less granular internal structure [4].

It is reported that clinically infected moose may experience diarrhea, emaciation, and potentially death. However, many moose survive heavy infections by isolating flukes within fibrous capsules [11]. Clinical symptoms reported are subtle or absent also in case of other liver fluke infection in wild cervids even morphologically there are observed hepatic chages [15, 16]. While the blood biochemistry of *P*.*fasciolaemorpha* has not been investigated, studies on other liver flukes such as *Fascioloides magna* have demonstrated significant alterations in liver enzymes (e.g., LDH, GLDH, ALT, AST, GGT), metabolic indicators (e.g., glucose, urea, triglycerides), and protein profiles (e.g., globulin, albumin/globulin ratio), reflecting hepatic dysfunction and systemic metabolic disturbances associated with liver trematodes [17, 18].

While *P*. *fasciolaemorpha* has been identified in moose and other wild ruminants, it remains considerably less documented than other liver flukes about its pathological effects and clinical impact. The aim of this study is to expand current knowledge by integrating the identification of the gross and histopathological examination of hepatic lesions alongside blood biochemical analysis and fecal examination. This comprehensive approach offers valuable insights into the pathogenic impact of this underexplored, less outspread trematode and its relevance to wildlife health and veterinary parasitology.

## 2. Materials and Methods

### 2.1. Study Area and Time

The study was carried out in different regions of throughout Latvia forests during hunting season between October and December in a five‐year period from 2017 to 2021. Climate is temperate with average temperature +6.40°C and relative humidity around 81% during hunting season, relatively even all over the country. Study area included several hunting territories in the Western, Middle, Norther and Eastern parts of Latvia, characterized by moderate annual precipitation and abundant surface water, including wetlands, swamps, and lakes, which provide suitable habitats for intermediate snail hosts. Based on data from the State Forest Service during study period the estimated moose population was approximately 23,000 individuals, with about 7000 harvested during hunting season with slight vary in numbers each year in Latvia.

### 2.2. Study Design

Study samples were collected from the 63 wild moose (*Alces alces*) of the different ages (young–< 1 year old and adult–more than 1 year old) and both genders (male and female) after they were hunted down in accordance with legislative acts of the republic of Latvia (Hunting law and regulations). Blood, fecal, liver samples were collected and whole liver was cut out within maximum 60 min after the moose were shot down and immediately transported at +4°C temperature for future examination to study liver biochemical, gross and histomorphological status in the wild moose invaded with trematode parasite *P*. *fasciolaemorpha*.

### 2.3. Morphological Examination

#### 2.3.1. Gross Examination

Each moose liver was post mortally examined at the Latvia University of Life Sciences and Technologies, Faculty of Veterinary medicine, Laboratory of Comparative pathology. The livers were examined in their entirety according to a standardized protocol, including systematic visual inspection and palpation of the whole organ. Serial transverse slices approximately 1–2 cm in thickness were made at regular intervals throughout the hepatic parenchyma to evaluate the parenchyma and major intrahepatic bile ducts.

Infection status of parafasciolopsosis was determined during gross liver examination based on characteristic trematode‐associated lesions, including bile duct thickening and parasite‐filled cavities. Bile ducts and associated cavities were longitudinally opened and manually explored to assess bile duct wall thickness, fibrous capsule formation, and intraluminal contents, including adult trematodes and necrotic detritus. All lesions were incised to confirm the presence or absence of adult *P*. *fasciolaemorpha*.

Parasites were identified by their size and morphological characteristics according to Filip, Pyziel and Demiaszkiewicz [4]. Individual parasites were not counted because reliable enumeration was not feasible due to aggregation, fragmentation, and partial degradation of trematodes within bile duct cavities and parasite‐associated detritus, resulting in non‐reliable parasite counts. Therefore, infection intensity in the strict parasitological sense was not assessed. Instead, trematode‐associated gross hepatic lesions were evaluated using a three‐grade semi‐quantitative scoring system adapted from 5 score system described by Zafra et al. [19], according to the trematode associated macroscopic lesions such as bile duct lesions, parasite caused cavities and divided in groups as follows: 0–no gross pathology changes, mild–< 10% liver affected, moderate 10%–30% liver infected and severe > 30% liver affected [19].

#### 2.3.2. Histological Examination

The collected liver tissue samples of each moose were immediately fixed in 10% neutral‐buffered formalin for histological examination. Samples were processed using standard procedures, embedded in paraffin blocks, sectioned to 4 µm thick slides using a semi‐automatic microtome and stained with hematoxylin and eosin (H&E) [20] as well as Masson’s trichrome [21]. Observations were made on light microscope Zeiss Axiolab 5. Histopathological changes were identified and quantified in three randomly selected microscopic fields at magnifications of × 50, × 100, × 200, and × 400 from both infected and uninfected animals. Masson’s trichrome staining was used to visualize connective tissue and measure the width of fibrous tissue capsules with Carl Zeiss Labscope lnk microscopic program. All photomicrographs were taken with Axiocam 208 color Zeiss camera.

### 2.4. Blood Biochemical Examination

Blood samples for biochemical examination were taken for 38 animals from 63 moose jugular vein collected in a one 2 mL size clot activator tube after the animal had fallen, delivered to the Latvia University of Life Sciences and Technologies Veterinary Clinics laboratory and analyzed within 24–48 h. The blood biochemistry was performed by Mindray BS‐200 analyzer to measure the blood chemistry parameters such us alanine aminotransferase (ALAT), aspartate aminotransferase (ASAT), gamma glutamyltransferase (GGT), total bilirubin (TBIL), alkaline phosphatase (ALP), total protein (TP), amylase, urea, creatinine, creatine phosphokinase (CK) and lactate dehydrogenase (LDH).

### 2.5. Coprological Examination

Fecal samples were directly taken for 39 animals from 63 moose rectum, placed in the plastic containers and immediately delivered refrigerated to the Latvia University of Life Sciences and Technologies, Parasitology laboratory for coprological examination. Coprological examination was performed for supportive purposes only and was not applied systematically to all animals, as infection status was determined based on gross liver examination. Each sample was screened using multiple diagnostic methods to detect a broad spectrum of parasitic infections, including trematodes, gastrointestinal nematodes, and lungworms. Ten grams of feces were examined for the presence of trematodes by standard sedimentation method as described by Hansen and Perry [22] and eggs of *P*. *fasciolaemorpha* were recognized by their characteristic morphological features according to Filip et al. [4]. For the presence of gastrointestinal nematodes, McMasters method was used [23]. The Baermann technique was apply to isolate larvae to diagnose lungworm infections [24].

### 2.6. Statistical Data Analysis

The data distribution of the blood examination was assessed by inspection of the normal Q‐Q plot and with the Shapiro‐Wilk test. Data homogeneity was assessed with the Levene test. For normally distributed data, mean values and standard deviations were reported. In contrast, the median and interquartile range (IQR) were used to summarize non‐normally distributed data. The Mann‐Whitney *U* test was used to compare multiple parameters of blood parameters between infected and non‐infected individuals. The chi‐squared test was used to test differences in histological changes between young and adult moose.

To account for the repeated histological evaluations conducted on three samples per individual, which introduced random variability due to non‐independence of measurements, generalized linear mixed models (GLMMs) were employed. These models were used to investigate the relationship between parasite infection (binary predictor: 1‐Yes, 0‐No) and the following binary histological outcomes: bile duct proliferation, portal fibrosis, inflammatory cell infiltration (portal), inflammatory cell infiltration (multifocal), and bile duct mucous gland hyperplasia.

Each outcome was modeled separately, with a logit link function applied to accommodate the binary nature of the response variables, fitting a binomial distribution. All statistical analyses were performed using Jamovi (version 2.5), with statistical significance determined at a threshold of *p* < 0.05.

## 3. Results

### 3.1. Gross Pathomorphological Changes

**FIGURE 1:**
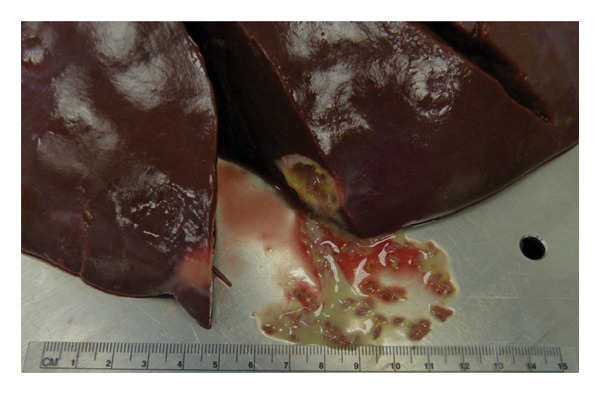
Gross appearance of the liver from an european moose infected with *Parafasciolopsis fasciolaemorpha*. Light‐gray foci are visible externally, and the cut section reveals cavities containing gray capsules with parasites and debris.

Study showed that totally from all examined 63 moose liver trematode *P*. *fasciolaemorpha* was found in 15 moose (24%) of which seven were young and eight were adults. Infected animals were identified across all regions of Latvia, with a higher number originated from eastern Latvia, which also contributed the largest number of examined animals, reflecting the distribution of the available material. Liver gross examination of infected animals showed thickening of the bile ducts, multiple cavities filled with trematodes *P. fasciolaemorpha* and yellowish gray cellular detritus. The thickened bile ducts and parasite filled cavities appeared on liver surface as light grey, round, 5–30 mm big spots (Figure 1). The capsule of the livers was crusted and uneven with white grainy sediment. In cross section of the bile ducts and cavities there were found multiple leaf‐shaped trematodes, approximately 5 mm long and 2 mm wide with two suckers (oral sucker and acetabulum) in the anterior end of the body and sharply narrowed posterior part of body which in terms of features correspond to a parasite *P*. *fasciolaemorpha.*

Trematode‐associated gross hepatic lesions ranged from mild to severe among the examined moose. Mild lesions were characterized by infrequent bile duct thickening and limited parasite‐filled cavities, whereas moderate and severe lesions showed more extensive hepatic involvement. Most of the young and adult animals had mild and moderate gross lesions of the liver invaded with *P*. *fasciolaemorpha.* Five young moose and three adult ones had mild liver changes, that is less than 10% of the organ affected with bile thickening and parasite filled cavities, four adult animals had moderate changes that is 10%–30% of the organ affected, but only two young and one adult moose had severe lesions, which affected more than 30% of the liver. For the rest of the study involved 48 moose (15 young and 33 adults) with no parasite presence there were no liver changes discovered.

### 3.2. Histopathological Changes

**FIGURE 2:**
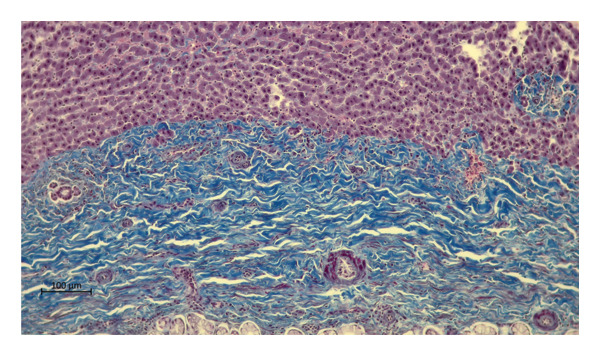
Photomicrograph of the Masson’s trichrome stained section from the *Parafasciolopsis fasciolaemorpha* infected liver with connective tissue (blue fibrils) capsule around parasite filled cavity. Magnification × 100.

Histopathological changes in the livers of moose naturally infected with the trematode *P*. *fasciolaemorpha* was investigated to characterize and compare the frequency and severity of hepatic lesions in infected versus uninfected individuals, with particular attention to inflammatory, fibrotic, and biliary tract alterations. The histopathological investigation displayed such lesions as portal fibrosis, inflammatory cell infiltration in portal areas, multifocal inflammatory cell infiltration in the parenchyma, bile duct proliferation and bile duct mucous gland hyperplasia. Macroscopically, liver cavities containing parasites were surrounded by a thick connective tissue capsule, which microscopically measured approximately 406.36 µm in width (Figure 2).

Histologically liver changes were assessed by counting microscopic fields with lesions mentioned above. Randomly chosen 3 microscopic fields in × 50, × 100, × 200, and × 400 of each animal liver samples were examined, total 45 from infected (15 animals) and 144 from uninfected moose (48 animals), to assess the impact of *P*. *fasciolaemorpha* infection on hepatic architecture and pathology. A summary of the lesions detected in the liver of moose with trematode *P*. *fasciolaemorpha* is provided in Table 1.

**TABLE 1:** Summary of the histological lesions detected in the european moose livers infected with the trematode *Parafasciolopsis fasciolaemorpha*.

Histological lesions	Number of the microscopic visual fields of the 15 infected and 48 not infected moose (%)			
	Infected moose with *Parafasciolopsis fasciolaemorpha* (*n* = 45)		Moose free from *Parafasciolopsis fasciolaemorpha* (*n* = 144)	
	7 young moose (*n* = 21)	8 adult moose (*n* = 24)	15 young moose (*n* = 45)	33 adult moose (*n* = 99)
Portal fibrosis	14 (67%)	13 (54%)	15 (33%)	32 (32%)
Inflammatory cell infiltration portal	16 (76%)	21 (88%)	23 (51%)	48 (48%)
Inflammatory cell infiltration multifocal	7 (33%)	5 (21%)	13 (29%)	8 (8%)
Bile duct proliferation	11 (52%)	10 (42%)	4 (9%)	10 (10%)
Bile duct mucous gland hyperplasia	5 (24%)	3 (13%)	0 (0%)	2 (2%)

**FIGURE 3:**
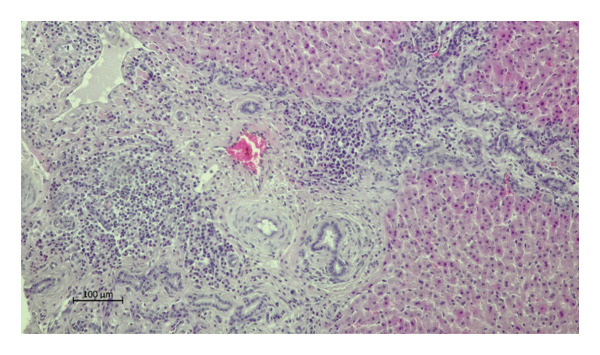
Photomicrograph of the H&E stained sections from the *Parafasciolopsis fasciolaemorpha* infected liver with portal inflammatory cell infiltrations accompanied with fibrosis and bilde duct proliferation. Magnification × 100.

**FIGURE 4:**
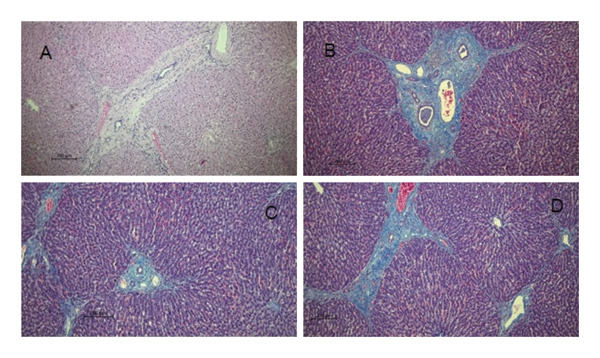
Photomicrographs of the sections from the *Parafasciolopsis fasciolaemorpha* infected livers with portal fibrosis. (A) H&E stained section with portal fibrosis between portal areas. Magnification × 50. (B) Masson’s trichrome stained section with mild fibrosis with minimal fibrous connective tissue (blue fibrils) formation. Magnification × 50. (C) Masson’s trichrome stained section with moderate fibrosis with connective tissue (blue fibrils) formation accompanied with inflammatory cell infiltrations and bilde duct proliferation. Magnification × 50. (D) Masson’s trichrome stained section with severe bridging fibrosis with fibrous septa (blue fibrils) between neighboring portal areas. Magnification × 50.

**FIGURE 5:**
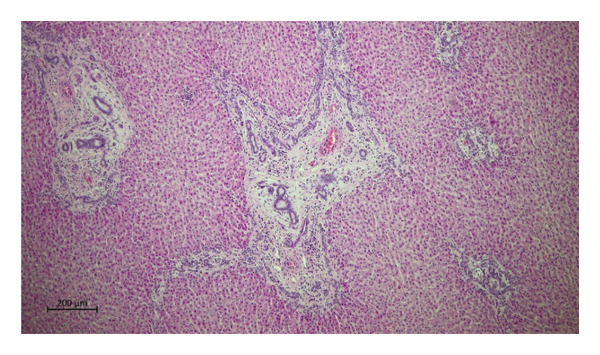
Photomicrograph of the H&E stained section from the *Parafasciolopsis fasciolaemorpha* infected liver with bilde duct proliferation accompanied by mild inflammatory cell infiltrations. Magnification × 50.

**FIGURE 6:**
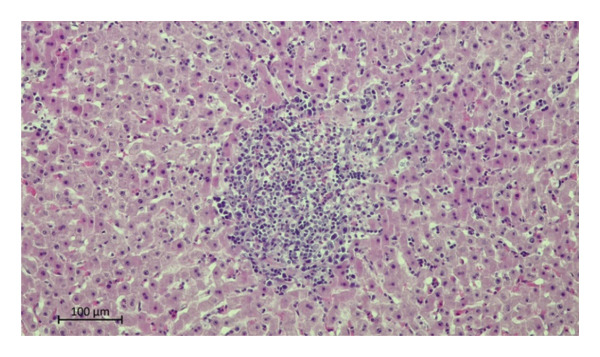
Photomicrograph of the H&E stained section from the *Parafasciolopsis fasciolaemorpha* infected liver with focal area of inflammatory cell infiltration. Magnification × 100.

**FIGURE 7:**
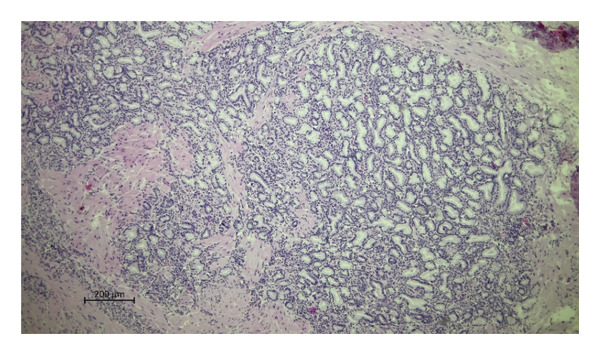
Photomicrograph of the H&E stained section from the *Parafasciolopsis fasciolaemorpha* infected liver with bile duct mucous gland hyperplasia. Magnification × 50.

Histopathological examination of the livers revealed a markedly higher prevalence of lesions in moose infected with *P*. *fasciolaemorpha* compared to uninfected controls. The most common histological lesion in moose infected with *P*. *fasciolaemorpha* was portal inflammatory cell infiltration (Figure 3), observed in 37 of 45 microscopic fields (82%). This lesion affected 16 of 21 microscopic fields of young moose (76%) and 21 of 24 microscopic fields of adults (88%), indicating a prominent and consistent immune response in the portal areas. Following portal inflammation in frequency was portal fibrosis (Figure 4(a)), identified in 27 of 45 microscopic fields of infected moose (60%) including 14 of 21 microscopic fields of young animals (67%) and 13 of 24 microscopic fields of adults (54%). The severity of portal fibrosis varied, presenting as mild fibrous expansion (Figure 4(b)), progressing to moderate fibrosis (Figure 4(c)), and in more advanced cases, forming bridging fibrous septa between neighboring portal areas (Figure 4(d)). This suggests that chronic inflammation may have progressed to fibrotic remodeling in a considerable proportion of cases. Biliary changes such as bile duct proliferation (Figure 5) was detected in 21 of 45 microscopic fields of infected moose (47%), with 11 of 21 microscopic fields of young moose (52%) and 10 of 24 microscopic fields of adults (42%). Among the less frequent findings were multifocal inflammatory cell inflammation and bile duct mucous gland hyperplasia. Multifocal inflammation (Figure 6), observed in 12 of 45 microscopic fields (27%), from which it was present more frequently in young animals (7 of 21 microscopic fields, 33%) compared to adults (5 of 24 microscopic fields, 21%). The least common lesion was bile duct mucous gland hyperplasia (Figure 7), which was detected in 8 of 45 microscopic fields (18%) from which it occurred in 5 of 21 microscopic fields of young animals (24%) and 3 of 24 microscopic fields of adults (13%). However, despite minor numerical variation between age groups, the chi‐squared test revealed no statistically significant differences in histological changes between young and adult moose *p* > 0.05. Comparable hepatic lesions were occasionally noted in uninfected moose, though at substantially reduced frequencies. In uninfected animals, age‐stratified data are presented in Table 1 for completeness, whereas histological findings are discussed collectively to characterize background hepatic changes in the absence of detectable parasites. In uninfected moose portal inflammatory infiltration was present in 71 of 144 microscopic fields animals (49%), portal fibrosis in 47 microscopic fields (33%), and bile duct proliferation in 14 microscopic fields (10%). Multifocal inflammatory infiltration was observed in 21 microscopic fields of individuals (15%), and bile duct mucous gland hyperplasia was rare, detected in only 2 microscopic fields (2%). Hepatic lesions of the non‐infected animals suggest that some changes may occur independently of parasitic infection.

Results of Generalized Linear Mixed Models (GLMMs) are summarized in Table 2. Parasite infection was significantly associated with bile duct proliferation (odds ratio (OR) = 6.84, 95% CI: 3.12–14.96, *p* < 0.001), indicating a nearly seven‐fold increase in the odds of bile duct proliferation in infected individuals compared to uninfected individuals. Similarly, parasite infection was significantly associated with portal fibrosis (OR = 3.00, 95% CI: 1.50–5.99, *p* = 0.002) and portal inflammatory cell infiltration (OR = 4.56, 95% CI: 1.98–10.48, *p* < 0.001), with infected individuals showing 3 and 4.5 times higher odds, respectively, compared to uninfected individuals. In contrast, no significant association was observed between parasite infection and multifocal inflammatory cell infiltration (OR = 4.54, 95% CI: 0.54–38.38, *p* = 0.164) or bile duct mucous gland hyperplasia (OR = 18.72, 95% CI: 0.44–795.55, *p* = 0.126). While the odds ratios for these outcomes were elevated, the wide confidence intervals and non‐significant *p* values suggest high variability and insufficient evidence to confirm an association.

**TABLE 2:** Generalized linear mixed model (GLMM) results for the association between *Parafasciolopsis fasciolaemorpha* infection and histological outcomes.

Outcome	Predictor	B	OR	95% CI	Z	*p*
Bile duct proliferation	Intercept	−1.09	0.34	0.23–0.50	−5.48	< 0.001
	Infected (Yes)^∗^	1.92	6.84	3.12–14.96	4.81	< 0.001

Portal fibrosis	Intercept	−0.144	0.87	0.61–1.22	−0.82	0.415
	Infected (Yes)^∗^	1.10	3.0	1.50–5.99	3.11	0.002

Inflammatory cell infiltration portal	Intercept	0.77	2.17	1.43–3.28	3.64	< 0.001
	Infected (Yes)^∗^	1.52	4.56	1.98–10.48	3.57	< 0.001

Inflammatory cell infiltration multifocal	Intercept	−2.67	0.07	0.01–0.35	−3.24	0.001
	Infected (Yes)^∗^	1.51	4.54	0.54–38.38	1.39	0.164

Bile duct mucous gland hyperplasia	Intercept	−6.70	0.001	0.0001–0.21	−2.55	0.011
	Infected (Yes)^∗^	2.93	18.72	0.44–795.55	1.53	0.126

*Note:* B–regression coefficient (estimate), Z–Wald z‐statistic, *p*–*p* value.

Abbreviations: CI, confidance interval; OR, odds ratio.

^∗^reference value–uninfected.

### 3.3. Blood Biochemical Findings

Biochemical blood analysis was conducted to evaluate systemic changes in moose infected with *P. fasciolaemorpha* (Table 3). Among the parameters analyzed, statistically significant differences were observed only in amylase and urea. Infected individuals showed a significantly lower concentration of amylase (median: 13.3 U/L; *p* = 0.009; *r* = 0.535) and urea (median: 1.69 mmol/L; *p* = 0.010; *r* = 0.525) compared to the uninfected group, with large effect sizes. No significant differences were found in liver‐associated enzymes (ALAT, ASAT, GGT, ALP), bilirubin, total protein, creatinine, or muscle injury markers (CK, LDH).

**TABLE 3:** Biochemical parameters in european moose with and without *Parafasciolopsis fasciolaemorpha*.

Parameter	With parasite		Without parasite		*p*	*r*
	Median	Q1–Q3	Median	Q1–Q3		
ALAT (U/L)	29.3	21.9–35.6	31.3	18.0–52.4	0.505	NA
ASAT (U/L)	115.2	99.2–165.2	148.0	96.0–407.6	0.179	NA
GGT (U/L)	11.2	4.4–27.9	11.4	8.5–20.2	0.751	NA
TBIL (µmol/L)	0.4	0.1–0.7	0.9	0–3.8	0.308	NA
ALP (U/L)	150.0	108.5–318.5	233.1	70.3–341.8	0.775	NA
TP (g/L)	76.7	67.3–83.3	73.6	63.9–80.5	0.544	NA
AMYLASE (U/L)	13.3	3.9–19.5	18.9	15.3–51.3	0.009	0.535
UREA (mmol/L)	1.69	1.0–2.8	3.3	1.8–4.2	0.010	0.525
CREAT (µmol/L)	119.0	115.3–144.3	127.5	114.4–149.9	0.849	NA
CK (U/L)	876.5	524.3–673.3	553.4	317.7–4280.5	0.890	NA
LDH (U/L)	839.1	673.3–917.9	774.1	683.8–1395.6	0.559	NA

*Note:* Q1–Q3–the first and third quartile, *r*–effect size, ALAT–alanine aminotransferase, ASAT–aspartate aminotransferase, GGT–gamma glutamyltransferase, TBIL–total bilirubin, ALP–alkaline phosphatase, AMYLASE–amylase, UREA–urea, CREAT–creatinine, CK–creatine phosphokinase, LDH–lactate dehydrogenase.

Abbreviations: NA, not applicable; TP, total protein.

### 3.4. Parasitological Findings

**FIGURE 8:**
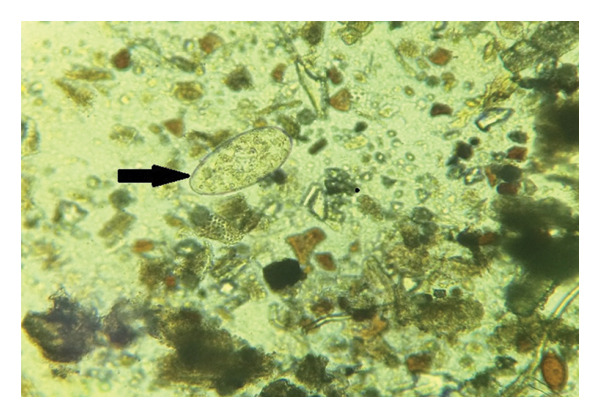
Egg of *Parafasciolopsis fasciolaemorpha* (arrow) observed under with light microscopy. Magnification × 100.

Among all examined fecal samples (*n* = 29), no *P. fasciolaemorpha* eggs were detected in samples from animals classified as uninfected based on gross liver examination. Of the remaining 10 animals in which adult parasite were identified in the liver, eggs were detected in nine fecal samples. One animal with trematode‐associated liver lesions did not shed detectable eggs in the examined fecal sample. Isolated eggs had light yellowish shells densely filled with yolk cells (Figure 8) Wild moose rectal fecal sample examination showed also presence of such parasite eggs as *Trichostrongylidae*, *Trichuris* spp., *Strongyloides* spp., *Protostrongylus* spp., *Moniezia* spp. and *Paramphistomum* spp. in both animal groups with the presence of the *P*. *fasciolaemorpha* in the moose liver and without it, but no of them are recognized as liver parasites.

## 4. Discussion

The present study provides a diverse evaluation of hepatic trematodiasis in wild moose naturally infected with *Parafasciolopsis fasciolaemorpha*, combining gross liver lesions and histological evaluation of parasite caused damages, complemented by blood biochemical analysis and fecal examination offering an overview of the host and parasite interaction in a wild cervid species. In both infected and uninfected animals were observed certain hepatobiliary changes that indicates that alterations even in reduced frequency may suggest effects unrelated to parasitic infection.

Parasitosis was determined by presence of the parasites with typical structure of *P. fasciolaemorpha* in the moose liver and macroscopic appearance of bile duct thickening, cavities filled with parasites and detritus, consistent with trematode‐induced hepatobiliary alterations. The gross lesions varied from mild to severe depending on the extent of trematode‐related hepatic involvement. The majority of infected animals, both young and adult, exhibited mild to moderate lesions, which may reflect early‐stage disease or effective host containment of hepatic involvement. Characteristic macroscopic changes were consistent with chronic trematode‐induced hepatobiliary damage, as similarly reported also in moose infected with parasite [11].

When interpreting the histological and biochemical findings, it should be acknowledged that, as with any diagnostic approach, some infected animals may not have been identified by gross liver examination, particularly in cases of early‐stage infection, low parasite load, or infections that had resolved prior to examination. Nevertheless, gross liver examination is expected to be highly specific for established trematode infection, as the observed lesions and parasite‐associated changes are unlikely to occur in the absence of infection. Any undetected infections would most likely reduce the observed differences between infected and uninfected groups rather than result in false‐positive findings, and this should be considered when interpreting the reported results.

Hepatic gross changes were accompanied by certain histomorphological changes. Comparable hepatic lesions were observed in both young and adult moose, suggesting a broadly similar pathological response to *P. fasciolaemorpha* infection across age groups. Whereas numerical differences between young and adult moose were observed for some histological parameters, these differences did not reach statistical significance, therefore, findings are discussed collectively, with age‐specific observations highlighted only where relevant. Statistically the most prominent hepatic pathomorphological lesion observed in the infected moose was bile duct proliferation followed by portal fibrosis and inflammatory cell infiltration supporting a strong association between *P. fasciolaemorpha* infection and the development of these hepatic changes. Bile duct proliferation identified alongside portal fibrosis was found in nearly half of the infected moose (47%). This change likely reflects regenerative or compensatory processes in response to ongoing biliary irritation or partial obstruction, suggesting sustained stimulation of the biliary epithelium. Comparable lesions have been also described in other studies that noted bile duct hyperplasia and disruptions in moose infected with *P. fasciolaemorpha* [16]. Biliary hyperplasia was observed also in some animals classified as uninfected which may reflect previous or resolved parasitic infections, chronic nonspecific biliary irritation, or other reasons not evaluated in this study. The portal inflammatory cell infiltration, affecting 82% of animals, indicating irritation of the hepatobiliary system. These findings are consistent with earlier descriptions in cervids which observed inflammatory infiltration and fibrotic changes specifically localized to the portal areas in moose naturally infected with *P. fasciolaemorpha* [16]. In contrast to observed predominating portal inflammatory cell infiltration observed in moose of our study, Filip‐Hutsch et al. [11] reported in 2019 an absence of inflammatory infiltration in hepatic connective tissue bands in moose fatally infected with *P. fasciolaemorpha*. This difference may reflect variation in host immune response, infection stage, or disease severity. It is possible that inflammatory infiltrates are more evident in sublethal infections with no evident clinical signs, whereas end‐stage or overwhelming infections may lead to tissue alterations dominated by fibrosis with limited active cellular inflammation. Portal fibrosis was observed in 60% of infected moose, with several cases demonstrating bridging fibrous septa between portal areas. Fibrotic changes observed in other studies showed more broader spectrum of fibrosis in moose infected with *P. fasciolaemorpha* from septal involvement to complete cirrhosis [11, 16] that likely reflect a later stage or higher intensity of infection. The case described by Filip‐Hutsch et al. in 2021 [16] that showed dominating fibrotic changes in the large liver regions in a fatal infection, is in contrast to our findings thus suggesting that portal fibrosis may develop progressively, is not limited to fatal pathology and may appear as early fibrotic phase of chronic hepatic disease.

Inflammatory and fibrotic hepatic lesions are also described in studies about liver fluke infections of other animal species. For instance, there is reported portal lymphocytic infiltration and periductal fibrosis as well as biliary proliferation in livers of sheep and cattle infected with Fasciola fluke infection [25–27] and periductal fibrosis and biliary hyperplasia in sheep naturally infected with *Dicrocoelium dendriticum* [28], suggesting that these lesions are common host response across ruminant species and trematode infections. More severe lesions have been described in infections with *Fascioloides magna*, which cause extensive liver parenchymal destruction of the fallow deer [29].

Lesions such as multifocal inflammatory cell infiltration and bile duct mucous gland hyperplasia did not show significant difference between infected and uninfected individuals. A slightly higher frequency of multifocal inflammatory cell infiltration was observed in young animals, however, this difference was not statistically significant and may reflect a mildly more widespread inflammatory response in immature individuals. In general, multifocal inflammatory cell infiltrates may indicate a more diffuse hepatic involvement in certain cases, which may reflect systemic immune response or more severe local tissue involvement. Regarding mucous gland hyperplasia, this relatively uncommon finding may represent a chronic adaptive response to bile duct irritation or partial obstruction, suggesting prolonged stimulation of biliary defense mechanisms. Similar lesions have been described by Kahl et al. [30] in sheep infected with liver and rumen flukes, characterized by pronounced bile duct epithelial hyperplasia, and mucosal gland proliferation were prominent features of chronic trematode‐induced hepatobiliary changes. Peribiliary gland hyperplasia is also reported in association with progressive biliary fibrosis and the occurrence of dysplastic lesions [31].

According to presence of hepatic lesions in animals classified as uninfected based on gross liver examination may be explained by several factors. Wild moose are exposed to a wide range of conditions affecting the liver, including previous parasitic infections, non‐hepatic parasites, ingestion of toxic plants, metabolic disturbances, and other environmental or physiological stressors, any of which may result in residual or nonspecific liver pathology. In addition, lesions observed in uninfected animals may represent sequelae of earlier infections that had resolved prior to examination, leaving persistent histopathological changes in the absence of detectable parasites.

In order to complement the morphological findings, biochemical blood analyses were performed to evaluate liver function and detect potential systemic effects of the infection. Biochemical analysis revealed significantly decreased levels of serum urea and amylase in infected moose compared to uninfected individuals, while other enzyme alterations in moose of different study groups were less pronounced. Thus, it showed that there were no overt signs of hepatic failure and indicated just mild functional impairment associated with parasitic liver lesions. Differences in serum urea and amylase levels between infected and uninfected moose suggest alterations in nitrogen metabolism and pancreatic function as a response to chronic parasite infection. The decreased urea concentration observed in infected moose may reflect diminished hepatic synthetic activity due to chronic inflammation or fibrosis, a pattern consistent with findings in naturally infected sheep, which exhibited significantly lower blood urea nitrogen levels, indicating impaired liver function [32]. According to studies of *Fascioloides magna* infection in red deer the urea values also were positively correlating with number of immature flukes and migratory lesions. Researchers also documented increased alanine aminotransferase (ALT) and globulin, lower values for albumin/globulin ratio and higher values of glucose for animals with hepatic fibrous capsules without the presence of migratory lesions [18] indicating more sever functional effects than in case of *P. fasciolaemorpha*. According to lower amylase activity it may suggest potential involvement and alteration of pancreatic or metabolic responses [33], though this parameter is less commonly evaluated in helminthic infections and may need further investigation.

Fecal analysis revealed the presence of *P. fasciolaemorpha* eggs in selected moose with parasite appearance in the liver, confirming active fluke infections and supporting the utility of coprological examination in diagnosing trematodiasis in wild cervids. The absence of detectable eggs in the fecal sample of one animal with confirmed parasite presence in the liver may reflect variability in egg excretion or limited passage of eggs into the intestine at the time of sampling. In addition to *P. fasciolaemorpha*, eggs of other gastrointestinal parasites were also detected such as *Trichostrongylidae*, *Trichuris* spp., *Strongyloides* spp., *Protostrongylus* spp., *Moniezia* spp. and *Paramphistomum* spp., suggesting concurrent infections that may further impact host health. Similar coinfections have been documented in wild ruminants [29, 34]. The detection of multiple parasite species highlights the complexity of parasite‐host interactions in wild populations and underscores the importance of need for parasitological monitoring.

Future studies should build upon this integrative approach, including advanced histological stains, detailed parasite investigations, and precise age determination as well as investigation to clarify the utility of biochemical markers in detecting and characterizing hepatic parasitosis in wildlife, particularly across different ruminant species. It would refine lesion characterization, clarify host and parasite interaction, and improve ability to monitor the health and conservation status of wild cervid populations.

## 5. Conclusions

This study demonstrates the integration of gross and microscopic liver pathology with blood biochemistry and coprological analysis in wild moose naturally infected with *Parafasciolopsis fasciolaemorpha*, revealing consistent hepatic lesions particularly bile duct alterations, portal inflammation and fibrosis as well as evidence of functional liver impairment. Biochemical findings, particularly reduced serum urea and amylase, suggest hepatic dysfunction, even in the absence of relevant clinical signs. Coprological analysis confirmed active trematode infection and frequent co‐infections with other parasites. Together, these findings provide foundational insight of liver fluke associated pathology in wild moose and underscore the value of integrating histology, biochemistry, and parasitology in wildlife health monitoring.

## Author Contributions

Baiba Bergmane: sample collection, data analysis, writing of the manuscript. Alina Klavina: identification of parasites. Maksims Zolovs: performing of the statistical analysis. Dace Gorbacevska: design of the study, supervision, data curation, review and editing of the manuscript.

## Funding

No funding form specific grant was received for this manuscript.

## Disclosure

All authors read and approved the final manuscript.

## Conflicts of Interest

The authors declare no conflicts of interest.

## Data Availability

The data that support the findings of this study are available from the corresponding author upon reasonable request.
